# Bis(tetra­phenyl­phospho­nium) tetra­iodidomanganate(II) acetone monosolvate

**DOI:** 10.1107/S1600536810006732

**Published:** 2010-02-27

**Authors:** Ehsan Jalilian, Sven Lidin

**Affiliations:** a Department of Enviromental and Material Chemistry, Arrhenius Laboratory, Stockholm University, 106 91 Stockholm, Sweden; b Polymer and Materials Chemistry, Lund University, 221 00 Lund, Sweden

## Abstract

The title compound, (C_24_H_20_P)_2_[MnI_4_]·(CH_3_)_2_CO, prepared from the reaction of manganese powder, iodine and tetra­phenyl­phospho­nium iodide in acetone shows a tetra­hedral complex anion [Mn—I = 2.6868 (5)–2.7281 (4) Å and I—Mn—I = 104.011 (13)–116.164 (15)°], two tetra­phenyl­phospho­nium cations and one mol­ecule of acetone.

## Related literature

For a general text on the luminescence of tetra­hedral Mn*X*_4_ (*X *= Cl, Br, I) complexes, see: Greenwood & Earnshaw (1984 ▶); Lee (1998 ▶). For structurally characterized Mn*X*_4_ complexes, see: Barber *et al.* (1980 ▶); Beagley *et al.* (1984 ▶, 1992 ▶); Davies *et al.* (1982 ▶); Godfrey *et al.* (1991 ▶); Howard *et al.* (1983 ▶); Hosseiny *et al.* (1980 ▶, 1981 ▶); McAuliffe *et al.* (1979 ▶, 1992 ▶); McAuliffe & Alkhateeb (1980 ▶). For the extinction correction, see: Becker & Coppens (1974 ▶).
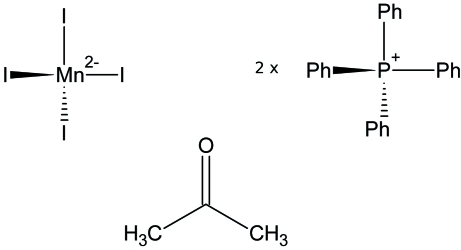


## Experimental

### 

#### Crystal data

(C_24_H_20_P)_2_[MnI_4_]·C_3_H_6_O
*M* *_r_* = 1299.4
Monoclinic, 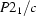

*a* = 19.5230 (4) Å
*b* = 14.9733 (3) Å
*c* = 17.6152 (4) Å
β = 105.161 (2)°
*V* = 4970.12 (19) Å^3^
*Z* = 4
Mo *K*α radiation
μ = 2.85 mm^−1^
*T* = 100 K
0.47 × 0.40 × 0.38 mm

#### Data collection

Oxford Diffraction Xcalibur3 with Sapphire-3 CCD detector diffractometer
Absorption correction: Gaussian (*CrysAlis RED*; Oxford Diffraction, 2008 ▶) *T*_min_ = 0.388, *T*_max_ = 0.480
181649 measured reflections
20267 independent reflections
12474 reflections with *I* > 3σ(*I*)
*R*_int_ = 0.055

#### Refinement

*R*[*F* ^2^ > 2σ(*F* ^2^)] = 0.034
*wR*(*F* ^2^) = 0.062
*S* = 1.17
20267 reflections
533 parameters
H-atom parameters constrained
Δρ_max_ = 2.03 e Å^−3^
Δρ_min_ = −1.59 e Å^−3^

### 

Data collection: *CrysAlis CCD* (Oxford Diffraction, 2008 ▶); cell refinement: *CrysAlis RED* (Oxford Diffraction, 2008 ▶); data reduction: *CrysAlis RED*; program(s) used to solve structure: *Superflip* (Oszlányi & Sütő, 2004 ▶); program(s) used to refine structure: *JANA2000* (Petříček & Dušek, 2000 ▶); molecular graphics: *DIAMOND* (Brandenburg, 1999 ▶); software used to prepare material for publication: *JANA2000*.

## Supplementary Material

Crystal structure: contains datablocks global, I. DOI: 10.1107/S1600536810006732/zs2027sup1.cif

Structure factors: contains datablocks I. DOI: 10.1107/S1600536810006732/zs2027Isup2.hkl

Additional supplementary materials:  crystallographic information; 3D view; checkCIF report

## Figures and Tables

**Fig. 1.**
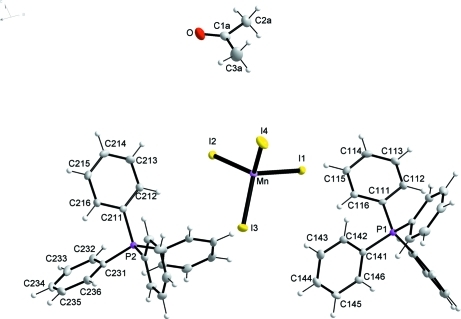
Molecular structure and atom-labelling scheme for the two cations, the complex anion and the acetone molecule of solvation in (I). Non-H atoms are shown as 50% probability displacement ellipsoids.

## supplementary crystallographic information

### Comment

Halide complexes with the composition [MnL_4_]^2-^ (L = Cl,Br,I) are well known for their luminescence properties (Greenwood & Earnshaw, 1984; Lee, 1998) but there are structural data for the tetrahalo complexes in the Cambridge Structural Database (Barber *et al.* (1980); Beagley *et al.*, 1984, 1992); Davies *et al.*, 1982; Godfrey *et al.* (1991); Howard *et al.*, 1983; Hosseiny *et al.*, 1980, 1981); McAuliffe *et al.*, 1979, 1992; McAuliffe & Alkhateeb, 1980), there is a surprising paucity of [MnI_4_]^2-^ structures. The compound presented here, the acetone solvate 2[(C_6_H_5_)_4_P^+^] [Mn I_4_]^2-^ . (CH_3_)_2_C═O (I), prepared from the reaction of manganese powder, iodine and tetraphenylphosphonium iodide in acetone under nitrogen, shows strong yellow luminescence. The strong absorbance of (I) in the visible spectrum is evident from the luminescence behaviour. Only very small crystals emit light homogeneously, while larger crystals emit primarily from the edges. The complex anion has tetrahedral stereochemistry with only small variations in Mn—I distances [range, 2.6868 (5)–2.7281 (4) Å] but has a somewhat larger variation in the I—Mn—I angles [104.011 (13)– 116.164 (15)°]. The counter cations are unexceptional with a narrow distribution of P—C and C—C distances [1.793 (3)–1.801 (3)Å and 1.369 (4)–1.409 (3)Å respectively], and C—P—C and (P/C)—C—C angles in the ranges 105.69 (12)–112.69 (12)° and 119.1 (2)–122.85 (19)° respectively.

### Experimental

Tetraphenylphosphonium iodide (3.34 mmol), iodine (6.86 mmol) and manganese powder (28.26 mmol) were mixed and heated under reflux in acetone (50 ml) under a nitrogen atmosphere. After 4 hours the solution became pale yellow. The mixture was filtered while hot and the solution was kept at -10°C. Centimeter-sized yellow crystals formed over the course of several months.

### Refinement

The structure wase solved by charge-flipping (Oszlanyi & Suto, 2004), giving the I, Mn, P and O positions, and a major number of the C positions. Subsequently the remaining C positions were found using difference Fourier analysis. All non-hydrogen positions were refined using full matrix least squares. The hydrogen atoms were located by geometrical methods and were allowed to ride, with C–H = 1.00 Å and *U*_eq_ = 1.2*U*_iso_(C).

### Crystal data

**Table:**

(C_24_H_20_P)_2_[MnI_4_]·C_3_H_6_O	*F*(000) = 2508
*M**_r_* = 1299.4	*D*_x_ = 1.736 Mg m^−^^3^
Monoclinic, *P*2_1_/*c*	Mo *K*α radiation, λ = 0.71069 Å
Hall symbol: -P 2ybc	Cell parameters from 57375 reflections
*a* = 19.5230 (4) Å	θ = 3.6–34.5°
*b* = 14.9733 (3) Å	µ = 2.85 mm^−^^1^
*c* = 17.6152 (4) Å	*T* = 100 K
β = 105.161 (2)°	Block, yellow
*V* = 4970.12 (19) Å^3^	0.47 × 0.40 × 0.38 mm
*Z* = 4	

### Data collection

**Table:**

Oxford Diffraction Xcalibur3 with Sapphire-3 CCD detector diffractometer	20267 independent reflections
Radiation source: Enhance (Mo) X-ray source	12474 reflections with *I* > 3σ(*I*)
graphite	*R*_int_ = 0.055
Detector resolution: 16.5467 pixels mm^-1^	θ_max_ = 34.6°, θ_min_ = 3.6°
ω scans	*h* = −30→30
Absorption correction: gaussian (*CrysAlis RED*; Oxford Diffraction, 2008)	*k* = −23→23
*T*_min_ = 0.388, *T*_max_ = 0.480	*l* = −28→27
181649 measured reflections	

### Refinement

**Table:**

Refinement on *F*^2^	Weighting scheme based on measured s.u.'s *w* = 1/[σ^2^(*I*) + 0.0004*I*^2^]
*R*[*F*^2^ > 2σ(*F*^2^)] = 0.034	(Δ/σ)_max_ = 0.027
*wR*(*F*^2^) = 0.062	Δρ_max_ = 2.03 e Å^−^^3^
*S* = 1.17	Δρ_min_ = −1.59 e Å^−^^3^
20267 reflections	Extinction correction: B–C type 1 Gaussian isotropic (Becker & Coppens, 1974)
533 parameters	Extinction coefficient: 3216
H-atom parameters constrained	

### Fractional atomic coordinates and isotropic or equivalent isotropic displacement parameters (Å^2^)

**Table:**

	*x*	*y*	*z*	*U*_iso_*/*U*_eq_	
I1	0.363127 (8)	0.413343 (12)	0.250596 (9)	0.01811 (5)	
I2	0.267877 (9)	0.620469 (12)	0.356895 (10)	0.01822 (5)	
I3	0.141259 (9)	0.494648 (12)	0.139458 (10)	0.02192 (5)	
I4	0.195916 (9)	0.328035 (14)	0.355201 (13)	0.03074 (7)	
Mn	0.237671 (18)	0.46300 (3)	0.27725 (2)	0.01406 (11)	
P1	0.47887 (3)	0.30247 (5)	0.01743 (4)	0.01217 (18)	
C111	0.53341 (11)	0.34965 (17)	0.10643 (13)	0.0123 (7)	
C112	0.60335 (12)	0.31820 (18)	0.13592 (14)	0.0163 (8)	
C113	0.64455 (12)	0.35154 (18)	0.20608 (15)	0.0175 (8)	
C114	0.61761 (12)	0.41635 (19)	0.24673 (14)	0.0192 (8)	
C115	0.54947 (12)	0.44842 (18)	0.21707 (14)	0.0169 (8)	
C116	0.50700 (12)	0.41493 (17)	0.14693 (13)	0.0134 (7)	
C121	0.45766 (12)	0.18827 (17)	0.03195 (14)	0.0138 (7)	
C122	0.39281 (13)	0.15420 (18)	−0.01153 (15)	0.0194 (8)	
C123	0.37516 (15)	0.06537 (19)	−0.00242 (16)	0.0245 (9)	
C124	0.42276 (15)	0.0112 (2)	0.04915 (16)	0.0245 (9)	
C125	0.48694 (14)	0.04433 (19)	0.09243 (16)	0.0231 (9)	
C126	0.50544 (13)	0.13267 (18)	0.08403 (15)	0.0190 (8)	
C131	0.52539 (12)	0.31146 (17)	−0.05770 (13)	0.0126 (7)	
C132	0.50406 (12)	0.25842 (17)	−0.12442 (14)	0.0163 (8)	
C133	0.53392 (13)	0.27192 (18)	−0.18677 (15)	0.0190 (8)	
C134	0.58487 (13)	0.33707 (18)	−0.18244 (15)	0.0198 (8)	
C135	0.60681 (12)	0.38938 (17)	−0.11562 (15)	0.0174 (8)	
C136	0.57672 (12)	0.37746 (18)	−0.05310 (14)	0.0157 (7)	
C141	0.39641 (12)	0.36202 (17)	−0.01352 (14)	0.0132 (7)	
C142	0.34925 (12)	0.36048 (17)	0.03393 (14)	0.0162 (8)	
C143	0.28478 (12)	0.40427 (18)	0.01055 (15)	0.0197 (8)	
C144	0.26611 (13)	0.44760 (18)	−0.06160 (16)	0.0215 (8)	
C145	0.31231 (13)	0.44827 (18)	−0.10941 (16)	0.0210 (8)	
C146	0.37791 (12)	0.40604 (17)	−0.08547 (14)	0.0160 (7)	
P2	0.06885 (3)	0.86274 (4)	0.17163 (4)	0.01173 (18)	
C211	0.08918 (12)	0.86045 (16)	0.27704 (13)	0.0130 (7)	
C212	0.15444 (12)	0.83129 (18)	0.32274 (14)	0.0175 (8)	
C213	0.16835 (13)	0.83290 (18)	0.40413 (15)	0.0208 (8)	
C214	0.11744 (14)	0.86422 (17)	0.43938 (15)	0.0190 (8)	
C215	0.05190 (14)	0.89340 (18)	0.39351 (15)	0.0198 (8)	
C216	0.03718 (13)	0.89155 (17)	0.31238 (14)	0.0173 (8)	
C221	0.00816 (11)	0.77516 (17)	0.13074 (14)	0.0132 (7)	
C222	−0.00761 (14)	0.70859 (18)	0.17697 (15)	0.0210 (8)	
C223	−0.04934 (15)	0.6372 (2)	0.14317 (16)	0.0258 (9)	
C224	−0.07579 (13)	0.63309 (19)	0.06201 (16)	0.0231 (9)	
C225	−0.06029 (13)	0.70026 (19)	0.01553 (15)	0.0210 (8)	
C226	−0.01808 (13)	0.77095 (19)	0.04895 (14)	0.0194 (8)	
C231	0.03084 (12)	0.97044 (17)	0.14318 (14)	0.0145 (7)	
C232	0.07276 (14)	1.04643 (18)	0.16375 (15)	0.0198 (8)	
C233	0.04151 (16)	1.13010 (19)	0.15066 (16)	0.0248 (10)	
C234	−0.03094 (17)	1.1383 (2)	0.11864 (16)	0.0276 (10)	
C235	−0.07273 (15)	1.0636 (2)	0.09981 (16)	0.0278 (10)	
C236	−0.04223 (13)	0.97791 (19)	0.11118 (15)	0.0200 (8)	
C241	0.14687 (12)	0.84220 (17)	0.13787 (14)	0.0146 (7)	
C242	0.17576 (12)	0.90571 (19)	0.09763 (14)	0.0172 (8)	
C243	0.23399 (13)	0.8829 (2)	0.06978 (15)	0.0217 (8)	
C244	0.26273 (12)	0.7991 (2)	0.08253 (15)	0.0214 (8)	
C245	0.23370 (12)	0.73475 (19)	0.12204 (15)	0.0202 (8)	
C246	0.17550 (12)	0.75620 (18)	0.14940 (14)	0.0168 (8)	
O	0.24790 (10)	0.42937 (14)	0.69284 (13)	0.0327 (7)	
C1a	0.27226 (14)	0.36418 (19)	0.66823 (16)	0.0236 (9)	
C2a	0.34963 (14)	0.3428 (2)	0.69304 (18)	0.0278 (10)	
C3a	0.22555 (17)	0.3008 (2)	0.6115 (2)	0.0435 (13)	
H112	0.622948	0.272114	0.106265	0.0195*	
H113	0.694022	0.32894	0.227737	0.021*	
H114	0.647665	0.439935	0.297714	0.023*	
H115	0.530696	0.495855	0.246171	0.0203*	
H116	0.457559	0.43782	0.125725	0.0161*	
H122	0.35887	0.193589	−0.04936	0.0233*	
H123	0.328352	0.041076	−0.033002	0.0294*	
H124	0.410536	−0.05286	0.055155	0.0294*	
H125	0.520496	0.004474	0.130205	0.0277*	
H126	0.552392	0.156264	0.114887	0.0228*	
H132	0.467466	0.210873	−0.127446	0.0196*	
H133	0.518477	0.23429	−0.235143	0.0228*	
H134	0.606015	0.346525	−0.227754	0.0238*	
H135	0.644339	0.435736	−0.112396	0.0208*	
H136	0.591736	0.415883	−0.005176	0.0189*	
H142	0.362153	0.327546	0.085093	0.0194*	
H143	0.251782	0.404718	0.045439	0.0237*	
H144	0.219177	0.478425	−0.079127	0.0258*	
H145	0.298402	0.479311	−0.161451	0.0252*	
H146	0.41145	0.407354	−0.119718	0.0192*	
H212	0.191288	0.809208	0.297139	0.021*	
H213	0.215144	0.811451	0.437299	0.025*	
H214	0.127754	0.865894	0.498038	0.0228*	
H215	0.015336	0.915796	0.419381	0.0237*	
H216	−0.009936	0.912134	0.279338	0.0208*	
H222	0.011209	0.711801	0.23544	0.0252*	
H223	−0.060463	0.588581	0.177031	0.0309*	
H224	−0.106036	0.581519	0.037283	0.0278*	
H225	−0.079881	0.697556	−0.042856	0.0253*	
H226	−0.006234	0.818975	0.015019	0.0233*	
H232	0.124971	1.040669	0.187768	0.0237*	
H233	0.071487	1.184884	0.164435	0.0298*	
H234	−0.052952	1.198984	0.109179	0.0331*	
H242	0.154964	0.967066	0.088784	0.0206*	
H243	0.254868	0.927988	0.040373	0.026*	
H244	0.305123	0.78388	0.063211	0.0257*	
H245	0.25478	0.673518	0.130557	0.0242*	
H246	0.153967	0.710293	0.177351	0.0202*	
H235	−0.12517	1.070113	0.07787	0.0333*	
H236	−0.072366	0.92337	0.0966	0.024*	
H21a	0.368944	0.34155	0.645773	0.0334*	
H22a	0.375065	0.389535	0.730538	0.0334*	
H23a	0.356701	0.283169	0.719425	0.0334*	
H31a	0.240116	0.300066	0.561103	0.0522*	
H32a	0.230395	0.23939	0.634612	0.0522*	
H33a	0.175029	0.320672	0.600884	0.0522*	

### Atomic displacement parameters (Å^2^)

**Table:**

	*U*^11^	*U*^22^	*U*^33^	*U*^12^	*U*^13^	*U*^23^
I1	0.01517 (7)	0.02419 (10)	0.01640 (8)	0.00176 (6)	0.00667 (6)	−0.00113 (7)
I2	0.02231 (7)	0.01323 (9)	0.01828 (8)	−0.00060 (6)	0.00380 (6)	−0.00199 (7)
I3	0.02058 (7)	0.02372 (11)	0.01732 (8)	−0.00178 (7)	−0.00243 (6)	−0.00117 (7)
I4	0.02084 (8)	0.02760 (12)	0.04608 (12)	−0.00157 (7)	0.01286 (8)	0.01617 (9)
Mn	0.01352 (15)	0.0144 (2)	0.01427 (17)	−0.00100 (14)	0.00358 (13)	−0.00023 (15)
P1	0.0119 (2)	0.0138 (3)	0.0108 (3)	0.0004 (2)	0.0029 (2)	−0.0005 (2)
C111	0.0120 (9)	0.0161 (14)	0.0088 (10)	−0.0011 (9)	0.0026 (8)	0.0011 (9)
C112	0.0125 (10)	0.0191 (15)	0.0172 (12)	0.0021 (9)	0.0037 (9)	0.0010 (10)
C113	0.0126 (10)	0.0189 (15)	0.0190 (12)	0.0008 (9)	0.0007 (9)	0.0048 (10)
C114	0.0161 (10)	0.0266 (16)	0.0129 (11)	−0.0072 (10)	0.0004 (9)	0.0012 (11)
C115	0.0194 (11)	0.0167 (14)	0.0157 (12)	−0.0010 (10)	0.0063 (9)	−0.0029 (10)
C116	0.0132 (9)	0.0137 (13)	0.0127 (11)	−0.0006 (9)	0.0022 (8)	0.0013 (10)
C121	0.0166 (10)	0.0136 (14)	0.0118 (11)	0.0005 (9)	0.0048 (8)	0.0000 (9)
C122	0.0214 (12)	0.0195 (15)	0.0153 (12)	−0.0028 (10)	0.0012 (9)	−0.0002 (10)
C123	0.0287 (14)	0.0222 (17)	0.0210 (13)	−0.0076 (11)	0.0038 (11)	−0.0017 (12)
C124	0.0389 (15)	0.0151 (16)	0.0226 (13)	−0.0028 (12)	0.0135 (12)	−0.0002 (11)
C125	0.0277 (13)	0.0193 (16)	0.0234 (14)	0.0053 (11)	0.0087 (11)	0.0062 (12)
C126	0.0192 (11)	0.0202 (16)	0.0177 (12)	0.0021 (10)	0.0050 (9)	0.0008 (11)
C131	0.0136 (9)	0.0122 (13)	0.0128 (11)	0.0029 (9)	0.0050 (8)	−0.0002 (9)
C132	0.0172 (11)	0.0144 (14)	0.0167 (12)	−0.0006 (9)	0.0033 (9)	−0.0041 (10)
C133	0.0204 (11)	0.0211 (16)	0.0160 (12)	0.0035 (10)	0.0059 (10)	−0.0043 (11)
C134	0.0213 (11)	0.0241 (16)	0.0168 (12)	0.0089 (11)	0.0098 (10)	0.0034 (11)
C135	0.0167 (10)	0.0137 (14)	0.0234 (13)	0.0022 (9)	0.0085 (9)	0.0014 (11)
C136	0.0175 (11)	0.0135 (14)	0.0161 (11)	0.0037 (10)	0.0044 (9)	0.0009 (10)
C141	0.0136 (10)	0.0126 (14)	0.0131 (11)	−0.0004 (9)	0.0030 (8)	−0.0012 (9)
C142	0.0160 (10)	0.0172 (15)	0.0150 (12)	−0.0003 (9)	0.0035 (9)	0.0008 (10)
C143	0.0143 (10)	0.0227 (16)	0.0226 (13)	0.0015 (10)	0.0057 (9)	−0.0030 (11)
C144	0.0175 (11)	0.0181 (15)	0.0273 (14)	0.0047 (10)	0.0029 (10)	0.0023 (12)
C145	0.0218 (12)	0.0179 (15)	0.0215 (13)	0.0025 (10)	0.0022 (10)	0.0079 (11)
C146	0.0171 (10)	0.0148 (14)	0.0156 (11)	−0.0008 (9)	0.0035 (9)	0.0012 (10)
P2	0.0110 (2)	0.0128 (4)	0.0119 (3)	0.0011 (2)	0.0038 (2)	0.0008 (2)
C211	0.0156 (10)	0.0106 (13)	0.0128 (11)	−0.0011 (9)	0.0036 (8)	0.0003 (9)
C212	0.0174 (11)	0.0181 (15)	0.0173 (12)	0.0030 (10)	0.0052 (9)	0.0012 (10)
C213	0.0204 (11)	0.0220 (16)	0.0167 (12)	0.0005 (11)	−0.0012 (9)	−0.0010 (11)
C214	0.0306 (13)	0.0125 (14)	0.0129 (11)	−0.0051 (10)	0.0037 (10)	−0.0019 (10)
C215	0.0265 (12)	0.0180 (16)	0.0181 (12)	0.0038 (10)	0.0117 (10)	−0.0004 (11)
C216	0.0180 (11)	0.0173 (15)	0.0174 (12)	0.0049 (10)	0.0059 (9)	0.0013 (10)
C221	0.0105 (9)	0.0142 (14)	0.0147 (11)	0.0006 (9)	0.0029 (8)	0.0008 (10)
C222	0.0282 (13)	0.0188 (16)	0.0152 (12)	−0.0034 (11)	0.0042 (10)	0.0003 (11)
C223	0.0355 (15)	0.0211 (17)	0.0208 (14)	−0.0101 (12)	0.0076 (11)	−0.0005 (12)
C224	0.0195 (12)	0.0223 (17)	0.0265 (14)	−0.0033 (11)	0.0039 (10)	−0.0079 (12)
C225	0.0209 (12)	0.0252 (16)	0.0144 (12)	−0.0006 (11)	0.0000 (9)	−0.0038 (11)
C226	0.0217 (12)	0.0227 (16)	0.0133 (12)	−0.0031 (10)	0.0038 (9)	0.0027 (11)
C231	0.0184 (11)	0.0130 (14)	0.0135 (11)	0.0048 (9)	0.0065 (9)	0.0036 (10)
C232	0.0232 (12)	0.0182 (15)	0.0214 (13)	0.0019 (11)	0.0119 (10)	0.0015 (11)
C233	0.0435 (16)	0.0137 (16)	0.0239 (14)	0.0003 (12)	0.0204 (12)	0.0016 (12)
C234	0.0486 (18)	0.0181 (17)	0.0209 (14)	0.0179 (13)	0.0176 (13)	0.0087 (12)
C235	0.0279 (14)	0.038 (2)	0.0185 (13)	0.0178 (13)	0.0083 (11)	0.0089 (13)
C236	0.0182 (11)	0.0233 (16)	0.0192 (12)	0.0049 (10)	0.0063 (9)	0.0024 (11)
C241	0.0115 (9)	0.0174 (14)	0.0140 (11)	0.0002 (9)	0.0017 (8)	−0.0028 (10)
C242	0.0169 (10)	0.0201 (15)	0.0144 (11)	−0.0008 (10)	0.0040 (9)	−0.0010 (10)
C243	0.0204 (11)	0.0294 (17)	0.0169 (12)	−0.0068 (11)	0.0076 (10)	−0.0012 (12)
C244	0.0126 (10)	0.0330 (18)	0.0194 (12)	0.0002 (10)	0.0056 (9)	−0.0088 (12)
C245	0.0161 (11)	0.0234 (16)	0.0195 (12)	0.0055 (10)	0.0020 (9)	−0.0068 (11)
C246	0.0144 (10)	0.0193 (15)	0.0163 (12)	−0.0003 (10)	0.0033 (9)	−0.0016 (10)
O	0.0273 (10)	0.0255 (13)	0.0414 (13)	0.0058 (9)	0.0019 (9)	−0.0016 (10)
C1a	0.0255 (13)	0.0204 (17)	0.0228 (14)	−0.0001 (11)	0.0027 (11)	0.0040 (12)
C2a	0.0274 (14)	0.0204 (17)	0.0352 (16)	0.0002 (12)	0.0072 (12)	−0.0012 (13)
C3a	0.0322 (17)	0.043 (2)	0.049 (2)	−0.0037 (15)	0.0009 (15)	−0.0165 (18)

### Geometric parameters (Å, °)

**Table:**

I1—Mn	2.7155 (4)	P2—C241	1.801 (3)
I2—Mn	2.7281 (4)	C211—C212	1.388 (3)
I3—Mn	2.6962 (4)	C211—C216	1.402 (4)
I4—Mn	2.6868 (5)	C212—C213	1.388 (4)
P1—C111	1.794 (2)	C212—H212	1.000
P1—C121	1.793 (3)	C213—C214	1.384 (4)
P1—C131	1.795 (3)	C213—H213	1.000
P1—C141	1.796 (2)	C214—C215	1.393 (3)
C111—C112	1.409 (3)	C214—H214	1.000
C111—C116	1.387 (4)	C215—C216	1.382 (4)
C112—C113	1.380 (3)	C215—H215	1.000
C112—H112	1.000	C216—H216	1.000
C113—C114	1.389 (4)	C221—C222	1.372 (4)
C113—H113	1.000	C221—C226	1.399 (3)
C114—C115	1.382 (3)	C222—C223	1.381 (4)
C114—H114	1.000	C222—H222	1.000
C115—C116	1.389 (3)	C223—C224	1.389 (4)
C115—H115	1.000	C223—H223	1.000
C116—H116	1.000	C224—C225	1.380 (4)
C121—C122	1.394 (3)	C224—H224	1.000
C121—C126	1.399 (3)	C225—C226	1.376 (4)
C122—C123	1.394 (4)	C225—H225	1.000
C122—H122	1.000	C226—H226	1.000
C123—C124	1.381 (4)	C231—C232	1.393 (4)
C123—H123	1.000	C231—C236	1.395 (3)
C124—C125	1.378 (4)	C232—C233	1.386 (4)
C124—H124	1.000	C232—H232	1.000
C125—C126	1.389 (4)	C233—C234	1.384 (4)
C125—H125	1.000	C233—H233	1.000
C126—H126	1.000	C234—C235	1.374 (4)
C131—C132	1.389 (3)	C234—H234	1.000
C131—C136	1.395 (4)	C235—C236	1.406 (4)
C132—C133	1.386 (4)	C235—H235	1.000
C132—H132	1.000	C236—H236	1.000
C133—C134	1.381 (4)	C241—C242	1.391 (4)
C133—H133	1.000	C241—C246	1.397 (4)
C134—C135	1.385 (4)	C242—C243	1.393 (4)
C134—H134	1.000	C242—H242	1.000
C135—C136	1.389 (4)	C243—C244	1.369 (4)
C135—H135	1.000	C243—H243	1.000
C136—H136	1.000	C244—C245	1.393 (4)
C141—C142	1.396 (4)	C244—H244	1.000
C141—C146	1.390 (3)	C245—C246	1.383 (4)
C142—C143	1.383 (3)	C245—H245	1.000
C142—H142	1.000	C246—H246	1.000
C143—C144	1.388 (4)	O—C1a	1.214 (4)
C143—H143	1.000	C1a—C2a	1.493 (4)
C144—C145	1.386 (4)	C1a—C3a	1.502 (4)
C144—H144	1.000	C2a—H21a	1.000
C145—C146	1.391 (3)	C2a—H22a	1.000
C145—H145	1.000	C2a—H23a	1.000
C146—H146	1.000	C3a—H31a	1.000
P2—C211	1.795 (2)	C3a—H32a	1.000
P2—C221	1.788 (2)	C3a—H33a	1.000
P2—C231	1.791 (3)		
			
I1—Mn—I2	104.011 (13)	C211—P2—C241	111.17 (11)
I1—Mn—I3	109.957 (15)	C221—P2—C231	111.43 (10)
I1—Mn—I4	106.881 (14)	C221—P2—C241	105.69 (12)
I2—Mn—I3	108.490 (14)	C231—P2—C241	112.69 (12)
I2—Mn—I4	116.164 (15)	P2—C211—C212	121.8 (2)
I3—Mn—I4	111.014 (13)	P2—C211—C216	117.60 (16)
C111—P1—C121	110.94 (11)	C212—C211—C216	120.6 (2)
C111—P1—C131	108.40 (11)	C211—C212—C213	119.7 (2)
C111—P1—C141	110.48 (11)	C211—C212—H212	120.1
C121—P1—C131	110.90 (12)	C213—C212—H212	120.1
C121—P1—C141	107.14 (11)	C212—C213—C214	120.0 (2)
C131—P1—C141	108.97 (11)	C212—C213—H213	120.0
P1—C111—C112	119.15 (19)	C214—C213—H213	120.0
P1—C111—C116	120.78 (16)	C213—C214—C215	120.3 (2)
C112—C111—C116	120.1 (2)	C213—C214—H214	119.9
C111—C112—C113	119.4 (2)	C215—C214—H214	119.9
C111—C112—H112	120.3	C214—C215—C216	120.3 (3)
C113—C112—H112	120.3	C214—C215—H215	119.9
C112—C113—C114	120.3 (2)	C216—C215—H215	119.8
C112—C113—H113	119.9	C211—C216—C215	119.1 (2)
C114—C113—H113	119.9	C211—C216—H216	120.4
C113—C114—C115	120.3 (2)	C215—C216—H216	120.4
C113—C114—H114	119.8	P2—C221—C222	121.44 (17)
C115—C114—H114	119.8	P2—C221—C226	118.3 (2)
C114—C115—C116	120.2 (2)	C222—C221—C226	120.0 (2)
C114—C115—H115	119.9	C221—C222—C223	120.3 (2)
C116—C115—H115	119.9	C221—C222—H222	119.8
C111—C116—C115	119.8 (2)	C223—C222—H222	119.8
C111—C116—H116	120.1	C222—C223—C224	119.8 (3)
C115—C116—H116	120.1	C222—C223—H223	120.1
P1—C121—C122	118.75 (18)	C224—C223—H223	120.1
P1—C121—C126	121.44 (18)	C223—C224—C225	119.9 (2)
C122—C121—C126	119.8 (2)	C223—C224—H224	120.0
C121—C122—C123	120.2 (2)	C225—C224—H224	120.0
C121—C122—H122	119.9	C224—C225—C226	120.5 (2)
C123—C122—H122	119.9	C224—C225—H225	119.8
C122—C123—C124	119.5 (2)	C226—C225—H225	119.8
C122—C123—H123	120.2	C221—C226—C225	119.5 (2)
C124—C123—H123	120.2	C221—C226—H226	120.2
C123—C124—C125	120.7 (3)	C225—C226—H226	120.2
C123—C124—H124	119.7	P2—C231—C232	119.37 (17)
C125—C124—H124	119.7	P2—C231—C236	119.4 (2)
C124—C125—C126	120.6 (2)	C232—C231—C236	120.6 (2)
C124—C125—H125	119.7	C231—C232—C233	119.5 (2)
C126—C125—H125	119.7	C231—C232—H232	120.3
C121—C126—C125	119.3 (2)	C233—C232—H232	120.3
C121—C126—H126	120.4	C232—C233—C234	120.4 (3)
C125—C126—H126	120.4	C232—C233—H233	119.8
P1—C131—C132	118.90 (19)	C234—C233—H233	119.8
P1—C131—C136	120.31 (19)	C233—C234—C235	120.3 (3)
C132—C131—C136	120.4 (2)	C233—C234—H234	119.8
C131—C132—C133	119.5 (2)	C235—C234—H234	119.8
C131—C132—H132	120.3	C234—C235—C236	120.4 (3)
C133—C132—H132	120.3	C234—C235—H235	119.8
C132—C133—C134	120.4 (2)	C236—C235—H235	119.8
C132—C133—H133	119.8	C231—C236—C235	118.8 (2)
C134—C133—H133	119.8	C231—C236—H236	120.6
C133—C134—C135	120.3 (3)	C235—C236—H236	120.6
C133—C134—H134	119.8	P2—C241—C242	122.85 (19)
C135—C134—H134	119.8	P2—C241—C246	116.8 (2)
C134—C135—C136	120.0 (2)	C242—C241—C246	120.3 (2)
C134—C135—H135	120.0	C241—C242—C243	119.3 (2)
C136—C135—H135	120.0	C241—C242—H242	120.4
C131—C136—C135	119.4 (2)	C243—C242—H242	120.4
C131—C136—H136	120.3	C242—C243—C244	120.2 (3)
C135—C136—H136	120.3	C242—C243—H243	119.9
P1—C141—C142	119.28 (18)	C244—C243—H243	119.9
P1—C141—C146	120.7 (2)	C243—C244—C245	120.9 (3)
C142—C141—C146	119.9 (2)	C243—C244—H244	119.6
C141—C142—C143	120.3 (2)	C245—C244—H244	119.6
C141—C142—H142	119.8	C244—C245—C246	119.5 (3)
C143—C142—H142	119.8	C244—C245—H245	120.3
C142—C143—C144	119.7 (3)	C246—C245—H245	120.3
C142—C143—H143	120.2	C241—C246—C245	119.8 (3)
C144—C143—H143	120.2	C241—C246—H246	120.1
C143—C144—C145	120.2 (2)	C245—C246—H246	120.1
C143—C144—H144	119.9	O—C1a—C2a	121.9 (2)
C145—C144—H144	119.9	O—C1a—C3a	121.3 (3)
C144—C145—C146	120.4 (2)	C2a—C1a—C3a	116.8 (3)
C144—C145—H145	119.8	H21a—C2a—H22a	109.5
C146—C145—H145	119.8	H21a—C2a—H23a	109.5
C141—C146—C145	119.4 (2)	H22a—C2a—H23a	109.5
C141—C146—H146	120.3	H31a—C3a—H32a	109.5
C145—C146—H146	120.3	H31a—C3a—H33a	109.5
C211—P2—C221	110.29 (12)	H32a—C3a—H33a	109.5
C211—P2—C231	105.66 (12)		
